# Salivary Metabolomics for Oral Squamous Cell Carcinoma Diagnosis: A Systematic Review

**DOI:** 10.3390/metabo12040294

**Published:** 2022-03-26

**Authors:** Kacper Nijakowski, Dawid Gruszczyński, Dariusz Kopała, Anna Surdacka

**Affiliations:** 1 Department of Conservative Dentistry and Endodontics, Poznan University of Medical Sciences, 60-812 Poznan, Poland; annasurd@ump.edu.pl; 2 Student’s Scientific Group, Department of Conservative Dentistry and Endodontics, Poznan University of Medical Sciences, 60-812 Poznan, Poland; dawid.j.gruszczynski@gmail.com (D.G.); dariuszkopalaa@gmail.com (D.K.)

**Keywords:** saliva, metabolomics, metabolome, metabolites, oral squamous cell carcinoma, oral cancer, head and neck squamous cell carcinoma, head and neck cancer, biomarkers, oncological diagnostics

## Abstract

Oral squamous cell carcinoma (OSCC) is the most common type of oral cancer in which the consumption of tobacco and alcohol is considered to be the main aetiological factor. Salivary metabolome profiling could identify novel biochemical pathways involved in the pathogenesis of various diseases. This systematic review was designed to answer the question “Are salivary metabolites reliable for the diagnosis of oral squamous cell carcinoma?”. Following the inclusion and exclusion criteria, nineteen studies were included (according to PRISMA statement guidelines). In all included studies, the diagnostic material was unstimulated whole saliva, whose metabolome changes were determined by different spectroscopic methods. At the metabolic level, OSCC patients differed significantly not only from healthy subjects but also from patients with oral leukoplakia, lichen planus or other oral potentially malignant disorders. Among the detected salivary metabolites, there were the indicators of the impaired metabolic pathways, such as choline metabolism, amino acid pathways, polyamine metabolism, urea cycle, creatine metabolism, glycolysis or glycerolipid metabolism. In conclusion, saliva contains many potential metabolites, which can be used reliably to early diagnose and monitor staging in patients with OSCC. However, further investigations are necessary to confirm these findings and to identify new salivary metabolic biomarkers.

## 1. Introduction

Oral cancer, a subtype of head and neck cancer, refers to a group of neoplasms affecting lips, oral cavity and oropharynx [1]. It represents one of the most common cancers in the world, with 476,125 new cases and 225,900 deaths in 2020 [2]. Oral squamous cell carcinoma (OSCC) accounts for over 90% of all oral cancer cases. OSCC may arise de novo or from pre-existing oral lesions, such as lichen planus, leukoplakia, erythroplakia and oral submucosal fibrosis, collectively referred to as oral potentially malignant disorders. Moreover, non-healing mucosal ulcerations are linked with the development of OSCC [3,4]. The principal etiological factors for OSCC are tobacco use and alcohol consumption, which have a synergistic carcinogenic effect. In the case of alcohol, smoked tobacco and smokeless tobacco, the odds for OSCC occurrence may be more than 16 times higher compared to people without addictions [5]. In addition, infections of human-papillomavirus (HPV) and Epstein–Barr virus (EBV), poor oral hygiene, dietary and genetic factors are associated with oral cancer development [6,7,8].

Clinically, the most common sites for OSCC are the tongue, floor of the mouth and lips [9]. Although the oral cavity is a relatively accessible site for self-examination and medical inspection, some lesions at the early stage may remain unnoticed or ignored due to the nearly asymptomatic course. Therefore, about 50% of OSCC cases are detected at a late phase (in stages III or IV), which implies a worse prognosis and high mortality rate [10,11,12]. Furthermore, conventional biopsy followed by histopathological analysis is considered the gold standard for the diagnosis of OSCC. However, this diagnostic procedure involves several drawbacks, such as invasiveness, inaccurate sampling caused by tumour heterogeneity and is impractical for large population screening and periodic monitoring of treatment response. It also needs extensive experience and professional equipment [13,14]. Hence, novel, sensitive and less invasive diagnostic tools based on molecular markers are advisable.

Saliva is an important, complex biological fluid containing a broad spectrum of minerals, electrolytes, nucleic acids, proteins, peptides, hormones, enzymes, antimicrobial constituents and other molecules. It provides biomarkers of health and disease status and reflects not only oral cavity diseases, but also systematic conditions. Thus, saliva is referred to as the “mirror of the body”. Additionally, saliva collection is non-invasive, easy and free of stress, constituting a reliable diagnostic medium [15,16,17]. Salivary biomarkers seem to be attractive in oncological diagnostics, especially in oral cancers communicating indirectly with saliva [18].

Previous studies have focused on the analysis of the proteome and transcriptome in serum and saliva samples. Researchers are currently concentrating their attention on novel diagnostics of the metabolome, whose changes reflect the disturbances of the metabolic pathways caused by pathophysiological processes. As the substrates, intermediates and end products of biochemical reactions, the metabolites are small molecules with a molecular weight typically less than 1500 Da [19,20]. The main analytical techniques used for metabolomics investigations are nuclear magnetic resonance (NMR) spectroscopy and mass spectrometry (MS), in combination with gas chromatography (GC), capillary electrophoresis (CE) or high-performance liquid chromatography (HPLC) [21,22].

Extensive salivary metabolite profiling can identify novel biochemical pathways involved in the pathogenesis of various diseases. Salivary metabolomic analysis has provided essential information on oral diseases, especially periodontal disease and oral cancer, as well as on systemic conditions, such as different types of cancer, neurodegenerative disorders, diabetes mellitus, cardiovascular diseases and viral infections [23,24,25,26,27,28,29,30]. Consequently, salivary metabolites could be used as biomarkers for early detection of disease, predicting prognosis or evaluating response to the applied treatment. Moreover, metabolomics may be beneficial for the development of personalised medicine [31]. However, the search for possible markers should take into account possible differences in the composition of metabolites in saliva, which is secreted by the specific salivary glands [32].

The present systematic review was designed in order to answer the question “Are salivary metabolites reliable for the diagnosis of oral squamous cell carcinoma?”, formulated according to the PICO (“population”, “intervention”, “comparison”, “outcome”).

## 2. Results

**Figure 1:**
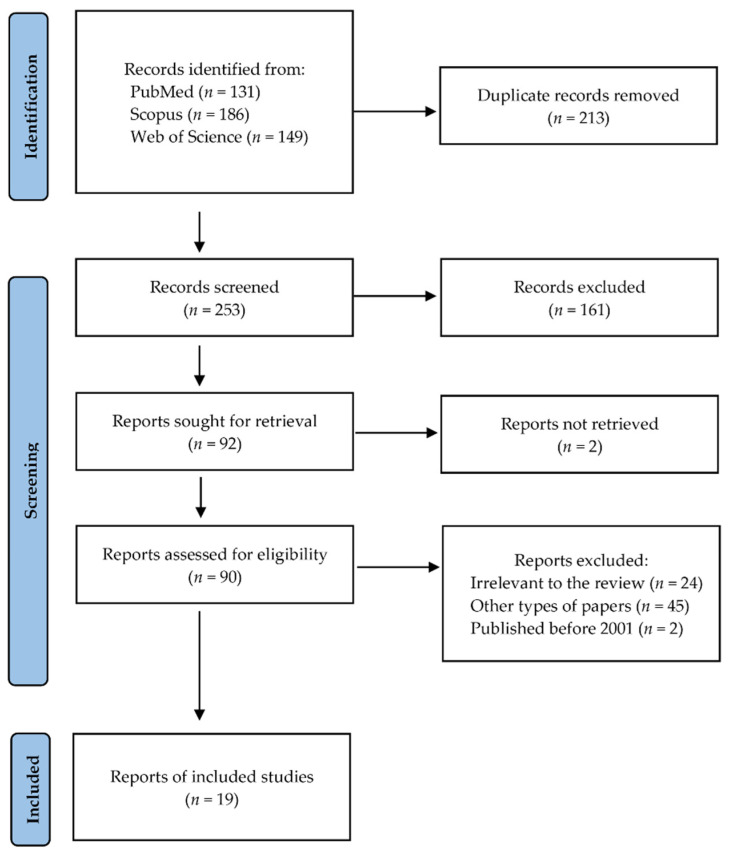
PRISMA flow diagram presenting search strategy.

Following the search criteria, our systematic review included nineteen studies, demonstrating data collected in six different countries from a total of 799 participants with diagnosed oral cancer (including 273 females, 506 males and 20 patients without reported gender). Figure 1 shows the detailed selection strategy of the articles. The inclusion and exclusion criteria are presented in the Section 4.

From each eligible study included in the present systematic review, we collected data about its general characteristics, such as year of publication and setting, involved participants, diagnosis and tumour–node–metastasis (TNM) staging, inclusion and exclusion criteria and smoking status (Table 1). Table 2 presents the detailed characteristics considering types of saliva, methods of collection, centrifugation, storing and laboratory analysis, as well as potential salivary metabolites for oral cancer. All of the studies took into consideration unstimulated whole saliva samples. Saliva centrifugation methods were rather heterogeneous but the most frequent method of sample storing was freezing at −80 °C. Additionally, predictive parameters for most discriminant metabolites from included studies were reported in Table 3.

**Figure 2:**
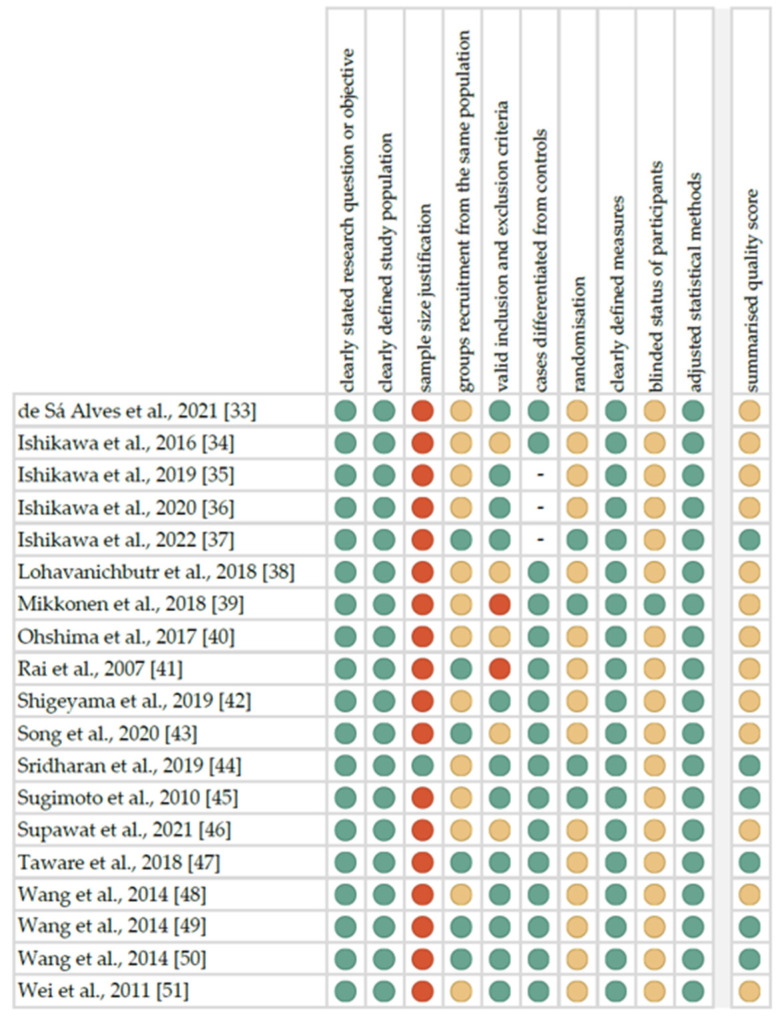
Quality assessment, including the main potential risk of bias (risk level: green—low, yellow—unspecified, red—high; quality score: green—good, yellow—intermediate, red—poor).

The summarised quality assessment for each study is reported in Figure 2. The most frequently encountered risks of bias were the absence of data regarding sample size justification (eighteen studies), blinding (eighteen studies) and randomisation (fifteen studies). Critical appraisal was summarised by adding up the points for each criterion of potential risk (points: 1—low, 0.5—unspecified, 0—high). Six studies (31.6%) were classified as having “good” quality (≥80% total score) and thirteen (68.4%) as “intermediate” (≥60% total score).

The level of evidence was assessed using the classification of the Oxford Centre for Evidence-Based Medicine levels for diagnosis [52]. All the included studies have the third or fourth level of evidence (in this 5-graded scale).

## 3. Discussion

Most of the included studies involved the detection of potential OSCC-specific metabolic markers compared to healthy subjects. The determined markers were diagnostic individually and in combination in patients with oral cancers of different stages.

Ohshima et al. [40] characterised the metabolic changes in saliva samples of Japanese patients with OSCC using capillary electrophoresis-mass spectrometry metabolome analysis. Among the potential twenty-five metabolites, choline showed the largest statistically significant difference between OSCC patients and healthy subjects. In addition to impaired choline metabolism, significant changes in other metabolic pathways were observed, e.g., branched-chain amino acid and aromatic amino acid pathways, polyamine metabolism, urea cycle, creatine metabolism and 3-hydroxybutyric acid metabolism. Virtually all observed changes in the salivary metabolome are associated with synthesis and degradation processes, which are reflected in the excessive proliferation of cancer cells. Moreover, Ishikawa et al. [34] explored salivary metabolites by profiling both saliva and tumour tissue samples for oral cancer screening. Among the detected metabolites, eighty-five were elevated in tumour tissue and forty-three in saliva, of which seventeen markers were common to both kinds of samples. Of these metabolites, based on the multivariate logistic regression modelling, the combination of S-adenosylmethionine and pipecolate demonstrated a high power to discriminate oral cancer patients from the controls. However, there were no significant differences in salivary metabolome according to the stage of OSCC.

In the latest study, Ishikawa et al. [37] evaluated the prognostic role of selected saliva metabolites. The authors randomly divided patients into training and validation groups in order to construct a Cox proportional hazards regression models. Two metabolites (5-hydroxylysine and 3-methylhistidine) were prognostically relevant for overall survival based on the training group. According to the analysis in the validation group, the statistically significant prognostic parameter for the OS was only 3-methylhistidine. For disease-free survival, N-acetylglucosamine appeared to be a potentially relevant prognostic factor based on the training group. However, no statistical significance was reached after adaptation to the model in the validation group. Kaplan–Meier survival curves were determined for the validation group. On this basis, OSCC patients with salivary concentrations of 3-methylhistidine above the median demonstrated significantly lower OS rates than those with the lower ones. Patients with increased salivary levels of N-acetylglucosamine had significantly higher DFS rates. The authors considered that salivary 3-methylhistidine (known as the indicator of sarcopenia status) might be an important factor in predicting the survival prognosis in OSCC patients.

Moreover, Sugimoto et al. [45] compared salivary metabolomes in patients with oral cancer, breast cancer, pancreatic cancer, periodontitis and healthy subjects, using capillary electrophoresis time-of-flight mass spectrometry. The multiple logistic regression model with nine potential metabolic markers for oral cancer patients had a good prediction determined by ROC analysis for differentiation from the controls, but lower than, e.g., the five-element model for pancreatic cancer.

In the study by Supawat et al. [46], salivary trimethylamine N-oxide and glycine were significantly higher in oral cancer patients. Similarly, Lohavanichbutr et al. [38] found the levels of glycine and proline to be significantly decreased in the saliva of OSCC patients. Additionally, four salivary metabolites, including glycine, proline, citrulline and ornithine, were related to early-stage OSCC (T1/T2) in both training and validation sets. However, there were no significant differences in the metabolome depending on the presence of lymph node metastases. Moreover, Mikkonen et al. [39] conducted multivariate discrimination function analysis to identify the combination of metabolites with maximal classification parameters for patients with head and neck squamous cell carcinoma. The highest discrimination power was achieved for fucose, glycine, methanol and proline. Decreased levels of amino acids indicate their overutilisation during carcinogenesis. Surfaces of cancer cells are affected by excessive fucosylation of glycoproteins which leads to uncontrolled tumour growth.

Interestingly, de Sá Alves et al. [33] conducted the first study focused on a group of Latin Americans with OSCC. The findings showed altered metabolic pathways, such as the malate–aspartate shuttle and the beta-alanine metabolism, as well as the Warburg effect. Above the threshold of AUC = 0.9, there were ten salivary metabolites as potential OSCC biomarkers: malic acid, lactose, catechol, 2-ketoadipic acid, maltose, methionine, urea, leucine, inosine and protocatechuic acid. Furthermore, Rai et al. [41] observed significantly lower levels of vitamins E and C in patients with oral cancer compared to healthy subjects. Given the contribution of these vitamins to protecting against free oxygen radicals, the authors recommend that oral cancer patients consider supplementing antioxidants to prevent cytotoxic effects and disease progression.

The specific group of metabolites seem to be the volatile organic metabolites. Volatile organic compounds are present in various biological fluids and can reflect the metabolic changes in response to pathological processes, such as inflammation, necrosis, degeneration or carcinogenesis. Using the method combining thin-film microextraction based on a ZSM-5/polydimethylsiloxane hybrid film coupled with gas chromatography-mass spectrometry, Shigeyama et al. [42] detected thirty-eight VOCs specifically from the OSCC group and thirty-five VOCs overlapping between OSCC patients and healthy subjects. Among the top ten, there were ketones, aldehydes and alcohols, which can be associated with the metabolic processes of cells during tumour formation, including oxidative reactions. The authors also proposed the diagnostic decision tree based on selected VOCs, suggesting that 2-pentanone over 2.11 × 10^5^ is less probable to be related to oral cancer diagnosis. Taware et al. [47] identified altered VOCs levels associated with the metabolic pathways, such as glycolysis or gluconeogenesis, pyruvate metabolism, glycerolipid metabolism, sulphur metabolism, selenoamino acid metabolism, taurine and hypotaurine metabolism, tyrosine metabolism and nicotinate and nicotinamide metabolism. The authors observed four volatile organic metabolites (1,4-dichlorobenzene, 1,2-decanediol, 2,5-bis1,1-dimethylethylphenol and E-3-decen-2-ol) with the highest specificity and sensitivity for class segregation between OC patients and control subjects.

For metabolic dysregulation caused by progressive exacerbation of premalignant lesions to OSCC, Song et al. [43] determined five crucial altered pathways such as aminoacyl tRNA biosynthesis, arginine/proline metabolism, arginine biosynthesis, lysine degradation and histidine metabolism. Unfortunately, similar significant differences in salivary metabolome between different stages of OSCC (from stage I to stage IV) were not found. The authors propose the combination of conductive polymer spray ionisation mass spectrometry (CPSI-MS) and machine learning (ML) as a potentially fast and non-invasive method of early detection of OSCC, with high accuracy of molecular diagnostics.

In three similar studies, Wang et al. [48,49,50] investigated potential salivary metabolic biomarkers to facilitate the early diagnosis of OSCC, using the ultra-performance liquid chromatography-electrospray ionisation-mass spectrometry (UPLC-ESI-MS). All selected metabolites differed in salivary concentrations between OSCC patients and healthy subjects. The two constructed combinations of biomarkers (choline, betaine, pipecolinic acid and L-carnitine; propionylcholine, N-acetyl-L-phenylalanine, sphinganine, phytosphingosine and S-carboxymethyl-L-cysteine) achieved satisfactory predictive parameters, such as accuracy, sensitivity and specificity in the discrimination of early stages of OSCC (I-II) from the controls. Excessive tumour cell proliferation requires an increased level of choline metabolism due to phosphorylation processes. Altered concentrations of betaine and L-carnitine are associated with downregulated fatty acid metabolism, as well as pipecolinic acid with upregulated lysine metabolism. Sphinganine and phytosphingosine are involved in the synthesis and metabolism of ceramide, which participates in the cellular signalling of apoptosis inducing. Considering single metabolic markers, L-leucine could have better predictive power for OSCC with T1/T2, and L-phenylalanine for OSCC with T3/T4. L-leucine stimulates protein synthesis and reduces protein breakdown, and L-phenylalanine is an essential precursor of tyrosine and catecholamines. When combined, both amino acids showed improved sensitivity and specificity for early detection of OSCC. The successfully presented innovative possibility of using salivary metabolites in a non-invasive and simple way can allow creating the clinical screening tool for the early diagnosis of OSCC.

Several studies have focused on the attempt to differentiate OSCC from other lesions of the oral mucosa, which may be considered oral potentially malignant disorders, based on changes in salivary metabolome. This is particularly important for oncological prevention and early detection of lesions. Wei et al. [51] selected the panel of five salivary metabolites (γ-aminobutyric acid, phenylalanine, valine, n-eicosanoic acid and lactic acid) and assessed their stratification value by ROC curves analyses. On this basis, 2–3-item combinations were created, the predictive power for OSCC was satisfactory in relation to patients with oral leukoplakia (OLK) and no changes in the oral mucosa. For OLK, the combination of valine, lactic acid and phenylalanine had higher parameters discriminating from OSCC (accuracy, sensitivity, specificity, positive predictive value). Higher levels of lactic acid and lower levels of amino acids are associated with increased glycolysis and impaired tricarboxylic acid cycle present in the cancer tissues during cell proliferation. The authors suggest that these findings could help complement the clinical differentiation of OSCC from OLK, improving prognosis with earlier detection. Similarly, Sridharan et al. [44] evaluated the clinical utility of salivary metabolites in oral leukoplakia and OSCC diagnosis. Both in OLK and OSCC patients, salivary metabolites such as sphinganine-1-phosphate, pseudouridine, 4-nitroquinolone-1-oxide, inositol 1,3,4-triphosphate, 2-phospho-glycerate, 1-methylhistidine, 2-oxoarginine, norcocaine nitroxide, L-isoleucine, gamma-aminobutyryllysine, L-homocysteic acid and ubiquinone were significantly upregulated. Compared to oral leukoplakia, a significant upregulation of D-glycerate-2-phosphate, estrone-3-glucoronide, 4-nitroquinolone-1-oxide, sphinganine-1-phosphate, 1-methylhistidine, inositol 1,3,4-triphosphate, d-glycerate-2-phosphate, 2-oxoarginine, norcocaine nitroxide and pseudouridine was observed for OSCC.

Ishikawa et al. [35] determined salivary metabolite markers to differentiate patients with oral squamous cell carcinoma and oral epithelial dysplasia (OSCC/OED) from those with persistent suspicious oral mucosal lesions (PSOML). From six significantly elevated metabolites in PSOML, ornithine, o-hydroxybenzoate and ribose 5-phosphate were selected in the multivariate logistic regression model, which together resulted in a high AUC value for the ROC curve discriminating from OSCC/OED patients. Ornithine, as the intermediate metabolite in the urea cycle, is the precursor of polyamines (e.g., putrescine), which are well known metabolic markers for various neoplasms. The Warburg effect is indicated by the reduction in intermediate metabolites in the pentose phosphate pathway, such as ribose 5-phosphate. According to the authors’ knowledge, it was the first study comparing both types of lesions on the oral mucosa.

Moreover, Ishikawa et al. [36] conducted another study to identify salivary metabolites for the discrimination of OSCC from oral lichen planus. Among fourteen metabolites significantly different between both groups, the combination of indole-3-acetate and ethanolamine phosphate showed a high AUC for discriminating OSCC from OLP. The first is produced by cancer tissue during the growth of malignant cells, and the second is the intermediate metabolite of the phospholipid metabolism involved in tumour progression. The authors suggest the potential of salivary metabolites for non-invasive screening of OSCC versus OLP.

Our systematic review collects and discusses the most recent information on the very fast-growing and promising diagnosis of salivary metabolites in oral cancer patients. Many metabolic markers still have an unknown significance for physiological and pathological processes. There is no doubt that saliva is a diagnostic material that is easy to collect and relatively simple to process for analytical diagnostics. However, changes in saliva composition, particularly metabolites, can be very dynamic and depend on many factors such as oral health status, the current microbiome activity and dietary habits. The stability of the salivary metabolome may also be affected by external factors such as collection or processing temperature and duration. Therefore, when testing such dynamic components, there is a risk of impaired diagnostic accuracy. Limitations of our systematic review include the heterogeneity of the included studies in terms of study design, clinical characteristics of patients with tumour progression, and laboratory methods. Not all authors explicitly stated the histopathological diagnosis of OSCC, although it can be assumed that the majority of these patients had the most common form of oral cancers. Similarly, despite the diversity of detected markers that disrupt numerous metabolic pathways, only some researchers reported statistical parameters to assess the diagnostic accuracy for these metabolites, which only allowed for the qualitative analysis of the extracted data.

## 4. Materials and Methods

### 4.1. Search Strategy and Data Extraction

A systematic review was conducted up to 7 February 2022, according to the Preferred Reporting Items for Systematic Reviews and Meta-Analyses (PRISMA) statement guidelines [53], using the databases PubMed, Scopus and Web of Science. The search formulas included:

- For PubMed: (((oral OR (head and neck)) AND (cancer OR carcinoma)) OR OSCC) AND saliva AND (metabolite OR metabolomics);
- For Scopus: TITLE-ABS-KEY((((oral OR “head and neck”) AND (cancer OR carcinoma)) OR OSCC) AND saliva AND (metabolite OR metabolomics));
- For Web of Science: TS = ((((oral OR (head and neck)) AND (cancer OR carcinoma)) OR OSCC) AND saliva AND (metabolite OR metabolomics)).

Records were screened by the title, abstract and full text by two independent investigators. Studies included in this review matched all the predefined criteria according to PICOS (“Population”, “Intervention”, “Comparison”, “Outcomes” and “Study design”), as shown in Table 4. A detailed search flowchart is presented in the Section 2. The study protocol was registered in International prospective register of systematic reviews PROSPERO (CRD42022312946).

### 4.2. Quality Assessment and Critical Appraisal for the Systematic Review of Included Studies

The risk of bias in each individual study was assessed according to the “Study Quality Assessment Tool” issued by the National Heart, Lung, and Blood Institute within the National Institute of Health [54]. These questionnaires were answered by two independent investigators, and any disagreements were resolved by discussion between them.

## 5. Conclusions

According to our systematic review, saliva contains many potential metabolites, which can be used reliably to early diagnose and monitor staging in patients with OSCC. However, further investigations are necessary to confirm these findings and to identify new salivary metabolic biomarkers.

## Figures and Tables

**Table 1:** General characteristics of included studies.

Author, Year	Setting	Study Group (F/M); Age	Control Group (F/M); Age	Diagnosis	Inclusion Criteria	Exclusion Criteria	Smoking Status	TNM Stages
de Sá Alves et al., 2021 [33]	Brazil	27 (8/19); 57 ± 13.87 (28–88)	41 (20/21); 57.34 ± 11.66 (31–86)	OSCC	OSCC: patients over 18 years of age concomitant with the diagnosis of OSCC; Ctrl: patients over 18 years of age, who wanted to participate in the research	OSCC: patients diagnosed with cancer anywhere on the body that had already undergone surgery, radiotherapy or chemotherapy; Ctrl: patients with some type of cancer during their lifetime	OSCC: 20 smokers; Ctrl: 8 smokers, 13 ex-smokers	I-15%, II-15%, III-22%, IV-48%
Ishikawa et al., 2016 [34]	Japan	24 (10/14); 72 (23–94)	44 (28/16); 68 (21–90)	OSCC (*n* = 21), malignant melanoma (*n* = 2), unknown (*n* = 1)	NR	OC: prior chemotherapy or radiotherapy; Ctrl: history of prior malignancy or autoimmune disorders	OC: 14 smokers; Ctrl: 9 smokers	I-21%, II-25%, III-33%, IV-21%
Ishikawa et al., 2019 [35]	Japan	OSCC: 6 (0/6); 63.5 (49–83), OED: 10 (4/6); 69.0 (57–81), PSOML: 32 (11/21); 62.5 (21–86)	-	OSCC, OED, PSOML	patients confirmed pathologically by open biopsy	prior chemotherapy or radiotherapy	NR	NR
Ishikawa et al., 2020 [36]	Japan	OSCC: 34 (14/20); 70.5 (29–87), OLP: 26 (21/5); 67.5 (34–98)	-	OSCC, OLP	OSCC patients confirmed pathologically by incisional open biopsy	prior chemotherapy or radiotherapy	NR	I-41.2%, II-26.5%, III-5.9%, IV-26.5%
Ishikawa et al., 2022 [37]	Japan	training group: 35 (15/20); 65.0 (26–89), validation group: 37 (19/18); 69 (23–94)	-	OSCC	prior curative treatment, such as radical surgery or chemoradiotherapy, OSCC patients confirmed pathologically by incisional open biopsy	prior non-curative treatment, such as palliative treatment or symptomatic treatment	training group: 2 smokers; validation group: 6 smokers	training group: 0 (CIS)-5.7%, I-45.7%, II-17.1%, III-8.6%, IV-22.9%; validation group: 0 (CIS)-2.7%, I-21.6%, II-21.6%, III-27.0%, IV-27.0%
Lohavanichbutr et al., 2018 [38]	USA	First set: 79 (23/56); <50—14 (17.7%), 50–59—24 (30.4%), 60–69—22 (27.8%), >70—19 (24.1%); Second set: 80 (17/63); <50—16 (20%), 50–59—37 (46.3%), 60–69—17 (21.3%), >70—10 (12.5%)	First set: 20 (8/12); <50—13 (65.0%), 50–59—4 (20.0%), 60–69—3 (15.0%), >70—0; Second set: 20 (5/15); <50—13 (65.0%), 50–59—3 (15.0%), 60–69—4 (20.0%), >70—0	OSCC	Ctrl: patients without OSCC who had oral surgery such as tonsillectomy at the same institutions where the OSCC patients were treated during the same period	NR	First set: 37 current smokers, 42 never/former smokers, Ctrl: 5 current smokers, 9 never/former smokers, 6 unknown; Second set: 28 current smokers, 51 never/former smokers, 1 unknown, Ctrl: 5 current smokers, 12 never/former smokers, 3 unknown	First set: T1/T2-50.6%, T3/T4-49.4%; Second set: T1/T2-68.0%, T3/T4-32.0%
Mikkonen et al., 2018 [39]	Brazil	8 (0/8); 61.7 ± 9.6 (52–76)	30; 54.4 ± 9.0 (42–74)	HNSCC: larynx (*n* = 5), oral cavity (*n* = 3)	NR	NR	HNSCC: 7 smokers; Ctrl: non-smokers	I-12.5%, II-0%, III-37.5%, IV-50%
Ohshima et al., 2017 [40]	Japan	22 (9/13); 68 ± 13	21 (13/8); 56 ± 8	OSCC	NR	OSCC: prior chemotherapy or radiotherapy, history of prior malignancy; Ctrl: history of mucosal diseases in the oral cavity, immunodeficiency, autoimmune disorders, hepatitis or HIV infection	NR	I-31.8%, II-31.8%, III-4.6%, IV-31.8%
Rai et al., 2007 [41]	India	50 (25/25); 17–50	24 (11/13); 18–50	OC	NR	NR	NR	III-100%
Shigeyama et al., 2019 [42]	Japan	12 (7/5); F: 60 ± 16.8, M: 64 ± 19	8 (1/7); F: 27, M:28.3 ± 10.3	OSCC	histologically diagnosed OSCC patients	OSCC: prior chemotherapy, radiotherapy, surgery or alternative remedies before sample collection; Ctrl: history of malignancy, immunodeficiency, underlying diseases	OSCC: 2 smokers, 1 ex-smoker; Ctrl: 1 smoker	I-41.7%, II-50.0%, III-0%, IV-8.33%
Song et al., 2020 [43]	China	discovery group: OSCC: 65 (30/35); 35–65, PML: 64 (30/34); 35–65, validation group: OSCC: 60 (30/30); 35–65, PML: 60 (30/30); 35–65	discovery group: 64 (30/34); 30–60, validation group: 60 (30/30); 30–60	OSCC, PML	NR	prior therapy	NR	discovery group: I-23.1%, II-32.3%, III-18.4%, IV-26.2%; validation group: I-23.3%, II-31.7%, III-18.3%, IV-26.7%
Sridharan et al., 2019 [44]	India	OSCC: 22 (4/18); 43 (39.5–54), OLK: 21 (2/19); 48 (38–54.5)	21 (7/14); 32 (27.5–45.5)	OSCC, OLK	OSCC: clinically and histopathologically confirmed OSCC; OLK: clinically diagnosed OLK; Ctrl: normal individuals without any oral lesions, tobacco habits and systemic illnesses	history of systemic illness and medications; history of therapy for OLK and OSCC and with recurrent oral lesions	OSCC: 2 smokers; OLK: 10 smokers	NR
Sugimoto et al., 2010 [45]	USA	OC: 69 (23/41/5 missing); 34–87 (59.5) (5 missing)	87 (27/42/18 missing); 20–75 (43) (2 missing)	OC	diagnosed with primary disease without metastasis	prior chemotherapy, radiotherapy, surgery or alternative therapy, history of prior malignancy, immunodeficiency, autoimmune disorders, hepatitis or HIV infection	NR	NR
Supawat et al., 2021 [46]	Thailand	15; 57.3 ± 8.9 (35–73)	10; 50.5 ± 10.7 (21–60)	OC	NR	Ctrl: history of cancer disease	OC: NR; Ctrl: non-smokers	NR
Taware et al., 2018 [47]	India	32 (13/19); 60 (36–82)	27 (12/15); 55 (33–75)	OC	minimum 18 years old patient with histopathological confirmation of malignant lesion	OC: anticancer therapeutic intervention; Ctrl: hypertension, diabetes, any medication during last 3 months	OC: 8 smokers; Ctrl: 8 smokers	NR
Wang et al., 2014 [48]	China	30 (5/25); 62	60 (25/35)	OSCC	clinical and histopathologic diagnosis	history of receiving medication, prior chemotherapy and radiotherapy	NR	I-23.3%, II-20%, III-6.7%, IV-50%
Wang et al., 2014 [49]	China	30 (5/25); 55 (29–72)	30 (5/25); 47 (25–69)	OSCC	clinical and histopathologic diagnosis	history of receiving medication and surgical operation, prior chemotherapy and radiotherapy	NR	I-13.3%, II-30%, III-10%, IV-46.7%
Wang et al., 2014 [50]	China	30 (5/25); 55 (29–72)	30 (5/25); 47 (25–69)	OSCC	clinical and histopathologic diagnosis	history of receiving medication and surgical operation, prior chemotherapy and radiotherapy	NR	I-13.3%, II-30%, III-10%, IV-46.7%
Wei et al., 2011 [51]	China	OSCC: 37 (11/26); 56 ± 11 (34–77), OLK: 32 (19/13); 60 ± 13 (34–80)	34 (21/13); 43 ± 14 (21–73)	OSCC, OLK	clinical and histopathologic diagnosis	history of receiving medication and treatment with topical or systemical steroids	OSCC: 10 smokers, OLK: 9 smokers, Ctrl: 6 smokers	I-24.3%, II-32.4%, III-16.2%, IV-27.1%

Legend: USA, the United States of America; F, female; M, male; -, not applicable; NR, not reported; Ctrl, control group; OSCC, oral squamous cell carcinoma; OC, oral cancer; OED, oral epithelial dysplasia; PSOML, persistent suspicious oral mucosal lesions; OLP, oral lichen planus; HNSCC, head and neck squamous cell carcinoma; PML, premalignant lesions; OLK, oral leukoplakia; CIS, carcinoma in situ.

**Table 2:** Detailed characteristics of included studies considering methods of collection and analysis of saliva.

Author, Year	Type of Saliva and Method of Collection	Centrifugation and Storing	Method of Analysis	Potential Discriminant Metabolites in Saliva
de Sá Alves et al., 2021 [33]	unstimulated whole saliva 3 mL collected in the plastic tubes, which were then hermetically closed, immersed in ice and transported within 1 h to the storage location	stored at −80 °C until analysis	GC-MS	22 metabolites: up: malic acid, maltose, methionine, inosine, protocatechuic acid, dihydroxyacetone phosphate, galacturonic acid, uracil, isocitric acid, ribose 5-phosphate, o-phospho-serine, indole-3-acetic acid, 2-ketoglutaric acid, pantothenic acid and spermidine; down: lactose, catechol, 2-ketoadipic acid, urea, leucine, margaric acid, palmitic acid and maleic acid
Ishikawa et al., 2016 [34]	unstimulated whole saliva 400 μL collected for 5–10 min in a 50 mL Falcon tube on ice; between 8 a.m. and 12 noon	immediately stored at −80 °C	CE-TOF-MS	among 43 significantly elevated metabolites, 17 metabolites also in tissue: up: 3-phosphoglyceric acid, pipecolate, spermidine, methionine, S-adenosylmethionine, 2-aminobenzamide, tryptophan, valine, hypoxanthine, glycylglycine, trimethylamine N-oxide, guanine, guanosine, taurine, choline, cadaverine, threonine
Ishikawa et al., 2019 [35]	unstimulated whole saliva 4–5 mL collected for 5–15 min into 50 mL Falcon tubes in a paper cup filled with crushed ice	immediately stored at −80 °C	CE-TOF-MS	6 metabolites: down: ornithine, carnitine, arginine, o-hydroxybenzoate, N-acetylglucosamine-1-phosphate and ribose 5-phosphate
Ishikawa et al., 2020 [36]	unstimulated whole saliva 3 mL collected for 5–10 min into 50 mL Falcon tubes in a paper cup filled with crushed ice	immediately stored at −80 °C	CE-TOF-MS	14 metabolites: up: trimethylamine N-oxide, putrescine, creatinine, 5-aminovalerate, pipecolate, N-acetylputrescine, gamma-butyrobetaine, indole-3-acetate, N1-acetylspermine, 2’-deoxyinsine, ethanolamine phosphate and N-acetylglucosamine, down: N-acetylhistidine and o-acetylcarnitine
Ishikawa et al., 2022 [37]	unstimulated whole saliva 3 mL collected for 5 min into 50 mL Falcon tubes in a paper cup filled with crushed ice	stored at −80 °C	CE-TOF-MS	for predicting overall survival: in the training group identified proline, carnitine, 5-hydroxylysine, 3-methylhistidine, adenosine, inosine and N-acetylglucosamine, in the validation group only 3-methylhistidine (HR = 1.711)
Lohavanichbutr et al., 2018 [38]	unstimulated whole saliva into 50 mL sterile conical centrifuge tube and transferred on ice to the laboratory within two hours	centrifuged at 1300× *g* at 4 °C for 10 min; stored at −80 °C	NMR and LC-MS	4 metabolites: citrulline and ornithine (only for T1/T2), proline and glycine
Mikkonen et al., 2018 [39]	unstimulated whole saliva sample collected into a sterile glass cup for 5 min; between 9 and 11 a.m.	centrifuged at 14,000 rpm for 6 min, stored at −20 °C	NMR spectroscopy	3 metabolites: up: 1,2 propanediol and fucose, down: proline
Ohshima et al., 2017 [40]	unstimulated whole saliva 5 mL collected for 5–10 min into 50 mL tubes, which were placed in a Styrofoam cup filled with crushed; at 8 a.m.	centrifuged at 2600× *g* for 15 min at 4 °C, and spun for a further 20 min in case of incomplete separation	CE-TOF-MS	25 metabolites: up: choline, p-hydroxyphenylacetic acid and 2-hydroxy-4-methylvaleric acid (*p*-value < 0.001), valine, 3-phenyllactic acid, leucine, hexanoic acid, octanoic acid, terephthalic acid, γ-butyrobetaine and 3-(4-hydroxyphenyl)propionic acid (*p*-value < 0.01), isoleucine, tryptophan, 3-phenylpropionic acid, 2-hydroxyvaleric acid, butyric acid, cadaverine, 2-oxoisovaleric acid, N6,N6,N6-trimethyllysine, taurine, glycolic acid, 3-hydroxybutyric acid, heptanoic acid and alanine (*p*-value < 0.05); down: urea (*p*-value < 0.05)
Rai et al., 2007 [41]	unstimulated whole saliva collected on ice	centrifuged and frozen at −20 °C until analysis	HPLC	vitamins E and C (*p*-value < 0.001)
Shigeyama et al., 2019 [42]	unstimulated whole saliva 2 mL, collected in a 10 mL glass bottle over a period of 5–10 min; for at least a period of 5 days between 7 and 10 a.m.	immediately stored at −80 °C	thin-film microextraction based on a ZSM-5/PDMS hybrid film coupled with GC-MS	among 27 volatile metabolites, 12 top metabolites: up: 3-heptanone, 1,3-butanediol, 1,2-pentanediol and 1-hexadecanol, down: ethanol, 2-pentanone, phenol, hexadecanoic acid, undecane, 1-octanol, butyrolactone and benzyl alcohol
Song et al., 2020 [43]	unstimulated whole saliva 500 μL, collected into an EP tube	centrifuged at 5000 rpm for 3 min, frozen at −80 °C until analysis	CPSI-MS	among 116 metabolites, top 10 metabolites: up: putrescine, cadaverine, thymidine, adenosine and 5-aminopentoate, down: hippuric acid, phosphocholine, glucose, serine and adrenic acid
Sridharan et al., 2019 [44]	unstimulated whole saliva was collected under aseptic conditions by drooling method in a collecting jar	immediately centrifuged and stored at −80 °C before analysis	UPLC-QTOF-MS	37 upregulated and 11 downregulated metabolites
Sugimoto et al., 2010 [45]	unstimulated whole saliva 5 mL for 5–10 min, spitted into 50 mL Falcon tubes, placed in a Styrofoam cup filled with crushed ice	centrifuged at 2600× *g* for 15 min at 4 °C and spun for 20 min in case of incomplete separation, transferred to two fresh tubes and frozen within 30 min	CE-TOF-MS	28 metabolites: up: pyrroline hydroxycarboxylic acid, leucine plus isoleucine, choline, tryptophan, valine, threonine, histidine, pipecolic acid, glutamic acid, carnitine, alanine, piperideine, taurine, C_4_H_9_N and C_8_H_9_N (*p*-value < 0.001); piperidine, alpha-aminobutyric acid, phenylalanine and C_6_H_6_N_2_O_2_ (*p*-value < 0.01); betaine, serine, tyrosine, glutamine, beta-alanine, cadaverine and C_5_H_14_N_5_, down: C_4_H_5_N_2_O_11_P (*p*-value < 0.05)
Supawat et al., 2021 [46]	unstimulated whole saliva collected on a sterile container kept in an ice pack	immediately stored at −20 °C until analysis	NMR spectroscopy	13 metabolites: up: trimethylamine N-oxide, taurine, glycine and aspartate, down: propionate, isobutyrate, fucose, cisaconitate, choline, trimethylamine N-oxide, methanol, acetoacetate and glycine
Taware et al., 2018 [47]	unstimulated whole saliva 2 mL collected in 10 mL sterilised glass vial with screw cap and immediately placed on ice; between 9 a.m. and 12 at noon	transported to the laboratory within 1 h and stored at −80 °C until analysis	HS-SPME-GC-MS	among 27 volatile metabolites, 15 top metabolites: 1,4-dichlorobenzene, 1,2-decanediol, 2,5-Bis1,1-dimethylethylphenol, propanoic acid (ethyl ester), E-3-decen-2-ol, acetic acid, propanoic acid, ethyl acetate, 2,4-dimethyl-1-heptene, 1-chloro-2-propanol, 1-chloro-2-butanol, 2-propenoic acid, 2,3,3-trimethylpentane, ethanol, 1,2,3,4-tetrachlorobutane
Wang et al., 2014 [48]	unstimulated whole saliva 3 mL kept on ice	centrifuged at 12,000 rpm for 20 min at 4 °C and frozen at −40 °C until analysis	UPLC-ESI-MS	2 metabolites: L-phenylalanine and L-leucine
Wang et al., 2014 [49]	unstimulated whole saliva 2 mL; between 9 and 11 a.m.	centrifuged at 12,000 rpm for 20 min at 4 °C and frozen at −40 °C until analysis	HILIC-UPLC-MS	4 metabolites: choline, betaine, pipecolinic acid and L-carnitine
Wang et al., 2014 [50]	unstimulated whole saliva 3 mL; between 9 and 11 a.m.	centrifuged at 12,000 rpm for 20 min at 4 °C and frozen at −40 °C until analysis	RP-UPLC-MS, HILIC-UPLC-MS	14 metabolites: up: lactic acid, hydroxyphenyllactic acid, N-nonanoylglycine, 5-hydroxymethyluracil, succinic acid, ornithine, hexanoylcarnitine and propionylcholine; down: carnitine, 4-hydroxy-L-glutamic acid, acetylphenylalanine, sphinganine, phytosphingosine and S-carboxymethyl-L-cysteine
Wei et al., 2011 [51]	unstimulated whole saliva; between 9 and 10 a.m.	centrifuged at 3500× *g* for 20 min at 4 °C and immediately stored at −80 °C until analysis	UPLC-QTOF-MS	among 41 metabolites, 5 top: gamma-aminobutyric acid, phenylalanine, valine, n-eicosanoic acid and lactic acid

Legend: GC-MS, gas chromatography-mass spectrometry; CE-TOF-MS, capillary electrophoresis time-of-flight mass spectrometry; NMR, nuclear magnetic resonance; LC-MS, liquid chromatography-mass spectrometry; HPLC, high-performance liquid chromatography; PDMS, polydimethylsiloxane; CPSI-MS, conductive polymer spray ionisation-mass spectrometry; UPLC-QTOF-MS, ultra-performance liquid chromatography coupled with quadrupole time of flight mass spectrometry; HS-SPME-GC-MS, headspace solid phase microextraction coupled with gas chromatography-mass spectrometry; UPLC-ESI-MS, ultra-performance liquid chromatography-electrospray ionisation-mass spectrometry; HILIC-UPLC-MS, ultra-performance liquid chromatography-mass spectrometry in hydrophilic interaction chromatography mode; RP-UPLC-MS, reversed-phase ultra-performance liquid chromatography-mass spectrometry; HR, hazard ratio.

**Table 3:** Determined predictive parameters for most discriminant metabolites from included studies.

Study	Most Discriminant Metabolites	AUC	−95% CI	+95% CI	Sensitivity [%]	Specificity [%]
de Sá Alves et al., 2021 [33]	Malic acid	0.981	-	-	-	-
	Lactose	0.964	-	-	-	-
	Catechol	0.947	-	-	-	-
	2-Ketoadipic acid	0.941	-	-	-	-
	Maltose	0.934	-	-	-	-
	Methionine	0.925	-	-	-	-
	Urea	0.925	-	-	-	-
	Leucine	0.923	-	-	-	-
	Inosine	0.922	-	-	-	-
	Protocatechuic acid	0.911	-	-	-	-
Ishikawa et al., 2016 [34]	3-Phosphoglyceric acid	0.767	0.635	0.899	-	-
	Pipecolate	0.755	0.637	0.873	-	-
	Spermidine	0.751	0.626	0.876	-	-
	Methionine	0.744	0.628	0.861	-	-
	S-adenosylmethionine	0.743	0.613	0.874	-	-
	S-adenosylmethionine + pipecolate	0.827	0.726	0.928	-	-
Ishikawa et al., 2019 [35]	Ribose 5-phosphate **	0.714	-	-	-	-
	Carnitine **	0.704	-	-	-	-
	Arginine **	0.689	-	-	-	-
	N-Acetylglucosamine1-phosphate **	0.682	-	-	-	-
	Ornithine **	0.676	-	-	-	-
	Ornithine + o-hydroxybenzoate + ribose 5-phosphate **	0.871	0.760	0.982	-	-
Ishikawa et al., 2020 [36]	5-Aminovalerate *	0.786	-	-	-	-
	Indole-3-acetate *	0.786	-	-	-	-
	Creatinine *	0.766	-	-	-	-
	Putrescine *	0.712	-	-	-	-
	N-Acetylglucosamine *	0.704	-	-	-	-
	Indole-3-acetate + ethanolamine phosphate *	0.856	0.762	0.950	-	-
Mikkonen et al., 2018 [39]	Fucose + glycine + methanol + proline	-	-	-	87.5	93.3
Shigeyama et al., 2019 [42]	2-Pentanone + undecane + 1,3-butanediol + hexadecanoic acid	-	-	-	95.8	94.0
Song et al., 2020 [43]	62 metabolites	0.992	0.978	1.000	90.0	98.3
Sugimoto et al., 2010 [45]	Alanine + choline + “leucine + isoleucine” + glutamic acid + C_8_H_9_N + phenylalanine + alpha-aminobutyric acid + serine	0.865	-	-	-	-
Taware et al., 2018 [47]	1,4-Dichlorobenzene	0.998	-	-	100.0	100.0
	1,2-Decanediol	0.939	-	-	100.0	80.0
	2,5-Bis1,1-dimethylethylphenol	0.913	-	-	90.0	80.0
	E-3-Decen-2-ol	0.889	-	-	80.0	80.0
Wang et al., 2014 [48]	L-Phenylalanine ^	0.695	0.560	0.830	84.6	61.7
	L-Leucine ^	0.863	0.747	0.979	84.6	81.7
	L-Phenylalanine + L-leucine ^	0.871	0.767	0.974	92.3	81.7
	L-Phenylalanine ^^	0.767	0.637	0.896	47.1	95.0
	L-Leucine ^^	0.852	0.748	0.956	82.4	80.0
	L-Phenylalanine + L-leucine^^	0.899	0.827	0.971	94.1	75.0
Wang et al., 2014 [49]	Choline ^	0.926	0.820	0.997	84.6	90.0
	Betaine ^	0.759	0.587	0.931	46.2	96.7
	Pipecolinic acid ^	0.994	0.981	1.000	92.3	96.7
	L-Carnitine ^	0.708	0.532	0.884	73.3	61.5
	Choline + betaine + pipecolinic acid + L-carnitine ^	0.997	0.989	1.000	100.0	96.7
	Choline ^^	0.898	0.781	1.000	82.4	96.7
	Betaine ^^	0.665	0.501	0.828	47.1	80.0
	Pipecolinic acid ^^	0.914	0.798	1.000	88.2	96.7
	L-Carnitine ^^	0.731	0.563	0.900	96.7	52.9
	Choline + betaine + pipecolinic acid + L-carnitine ^^	0.906	0.804	1.000	88.2	90.0
Wang et al., 2014 [50]	Propionylcholine ^	0.946	0.882	1.000	76.9	96.7
	S-carboxymethyl-L-cysteine ^	0.913	0.822	1.000	84.6	93.3
	Phytosphingosine ^	0.910	0.816	1.000	92.3	83.3
	Acetylphenylalanine ^	0.838	0.705	0.972	92.3	76.7
	Sphinganine ^	0.818	0.660	0.976	84.6	83.3
	Propionylcholine + acetylphenylalanine + sphinganine + phytosphingosine + S-carboxymethyl-L-cysteine ^	0.997	-	-	100.0	96.7
	Propionylcholine + acetylphenylalanine + sphinganine + phytosphingosine + S-carboxymethyl-L-cysteine ^^	0.971	-	-	86.7	94.1
	S-carboxymethyl-L-cysteine ^^	0.888	0.784	0.992	88.2	90.0
	Phytosphingosine ^^	0.875	0.776	0.973	76.5	83.3
	Lactic acid ^^	0.837	0.723	0.951	100.0	73.3
	Propionylcholine ^^	0.788	0.655	0.921	64.7	80.0
	Succinic acid ^^	0.786	0.658	0.914	88.2	66.7
Wei et al., 2011 [51]	Lactic acid	0.800	0.700	0.904	73.0	70.6
	Gamma-Aminobutyric acid	0.560	0.423	0.698	61.8	62.2
	Valine	0.810	0.706	0.911	82.4	75.7
	Phenylalanine	0.640	0.508	0.765	52.9	56.8
	n-Eicosadienoic acid	0.670	0.549	0.800	51.4	73.5
	Lactic acid + valine	0.890	0.813	0.972	86.5	82.4
	Lactic acid ***	0.820	0.724	0.918	73.0	75.0
	gamma-Aminobutyric acid ***	0.750	0.636	0.869	75.0	70.3
	Valine ***	0.830	0.736	0.925	78.1	75.8
	Phenylalanine ***	0.780	0.662	0.894	71.9	75.7
	n-Eicosadienoic acid ***	0.770	0.658	0.886	70.3	87.5
	Lactic acid + valine + phenylalanine ***	0.970	0.932	1.000	94.6	84.4

Legend: AUC, area under curve; CI, confidence interval; -, not reported; *, vs. oral lichen planus; **, vs. persistent suspicious oral mucosal lesions; ***, vs. leukoplakia; ^, OSCC I-II; ^^, OSCC III-IV.

**Table 4:** Inclusion and exclusion criteria according to the PICOS.

Parameter	Inclusion Criteria	Exclusion Criteria
Population	patients with oral cancer—aged from 0 to 99 years, both sexes	patients with other neoplasms
Intervention	not applicable	
Comparison	not applicable	
Outcomes	salivary metabolites as markers	other salivary components as markers
Study design	case-control, cohort and cross-sectional studies	literature reviews, case reports, expert opinion, letters to the editor, conference reports
	published after 2000	not published in English

## Data Availability

Data are available on request from the corresponding author. The data are not publicly available due to this is a systematic review (not an original article), so the database is in Excel and contains the data already displayed in most Tables in our manuscript.
